# Symptoms reported by gastrointestinal stromal tumour (GIST) patients on imatinib treatment: combining questionnaire and forum data

**DOI:** 10.1007/s00520-022-06929-3

**Published:** 2022-03-02

**Authors:** Dide den Hollander, Anne R. Dirkson, Suzan Verberne, Wessel Kraaij, Gerard van Oortmerssen, Hans Gelderblom, Astrid Oosten, Anna K. L. Reyners, Neeltje Steeghs, Winette T. A. van der Graaf, Ingrid M. E. Desar, Olga Husson

**Affiliations:** 1 Department of Medical Oncology, Netherlands Cancer Institute, Amsterdam, the Netherlands; 2 Department of Medical Oncology, Radboud University Medical Center, Nijmegen, the Netherlands; 3 Leiden Institute of Advanced Computer Science, Leiden University, Leiden, the Netherlands; 4 Dutch Patient Platform Sarcomas, Utrecht, the Netherlands; 5 Department of Medical Oncology, Leiden University Medical Center, Leiden, the Netherlands; 6 Department of Medical Oncology, Erasmus MC Cancer Institute, Erasmus University Medical Center, Rotterdam, the Netherlands; 7 Department of Medical Oncology, University Medical Center Groningen, University of Groningen, Groningen, the Netherlands; 8 Department of Surgical Oncology, Erasmus Medical Center, Rotterdam, the Netherlands; 9 Division of Clinical Studies, Institute of Cancer Research, London, UK

**Keywords:** Patient-reported outcomes, Patient forum, Social media mining, Symptom measurement, Tyrosine kinase inhibitors, Gastrointestinal stromal tumours

## Abstract

**Purpose:**

Treatment with the tyrosine kinase inhibitor (TKI) imatinib in patients with gastrointestinal stromal tumours (GIST) causes symptoms that could negatively impact health-related quality of life (HRQoL). Treatment-related symptoms are usually clinician-reported and little is known about patient reports. We used survey and online patient forum data to investigate (1) prevalence of patient-reported symptoms; (2) coverage of symptoms mentioned on the forum by existing HRQoL questionnaires; and (3) priorities of prevalent symptoms in HRQoL assessment.

**Methods:**

In the cross-sectional population-based survey study, Dutch GIST patients completed items from the EORTC QLQ-C30 and Symptom-Based Questionnaire (SBQ). In the forum study, machine learning algorithms were used to extract TKI side-effects from English messages on an international online forum for GIST patients. Prevalence of symptoms related to imatinib treatment in both sources was calculated and exploratively compared.

**Results:**

Fatigue and muscle pain or cramps were reported most frequently. Seven out of 10 most reported symptoms (i.e. fatigue, muscle pain or cramps, facial swelling, joint pain, skin problems, diarrhoea, and oedema) overlapped between the two sources. Alopecia was frequently mentioned on the forum, but not in the survey. Four out of 10 most reported symptoms on the online forum are covered by the EORTC QLQ-C30. The EORTC-SBQ and EORTC Item Library cover 9 and 10 symptoms, respectively.

**Conclusion:**

This first overview of patient-reported imatinib-related symptoms from two data sources helps to determine coverage of items in existing questionnaires, and prioritize HRQoL issues. Combining cancer-generic instruments with treatment-specific item lists will improve future HRQoL assessment in care and research in GIST patients using TKI.

**Supplementary Information:**

The online version contains supplementary material available at 10.1007/s00520-022-06929-3.

## Introduction

GISTs represent a rare (10–20 cases per 1,000,000/year) family of mesenchymal tumours arising anywhere along the gastrointestinal tract [1]. Treatment with tyrosine kinase inhibitors (TKIs) improves survival for patients with a gastrointestinal stromal tumour (GIST), both in the adjuvant and palliative setting, but is not without side-effects [2–4]. TKIs are the only effective systemic treatment for high-risk localized and advanced GISTs [5]. Specifically, imatinib has significantly changed the prognosis of non-resectable advanced or metastatic GIST patients: from a median overall survival of 14–18 up to 57 months [6]. TKIs are taken orally on a daily basis until progressive disease. Especially, imatinib is considered to be moderate to well tolerated, at least when compared to conventional chemotherapy [7]. Side-effects are seen in virtually all patients, with the most frequent being (periorbital) oedema, diarrhoea, fatigue, myalgia/musculoskeletal pain, and nausea [8].

Treatment-related side-effects or symptoms have a significant impact on health-related quality of life (HRQoL) and are an important aspect of HRQoL assessment. HRQoL and symptoms can be assessed using patient-reported outcome measures (PROMs), providing subjective assessments coming directly from the patient, without interpretation by health care professionals or anyone else [9]. The patient perspective is needed to create a more complete overview of treatment-related symptoms, as previous research has shown a gap between the reporting by clinicians and by patients, with clinicians under-reporting symptoms [10, 11]. Another resource of patient-reported data is social media, including patient forums, i.e. online communities where patients exchange information and experiences. Social media are increasingly recognized as sources for reports of patient experiences including symptoms [12]. The reports from social media are unselected, unsolicited, and unbiased, and indicate which symptoms have an impact on their health or daily life [13] without the burden of completing questionnaires. Furthermore, its data can also detect emerging issues that may not be mentioned in registration trials or are not covered in existing PROMs [14, 15].

Few studies have investigated patient-reported symptoms in patients with GIST using TKIs. In a qualitative study, 77 different symptoms were reported by GIST patients using TKIs [16]. In another interview study [17], GIST patients with metastatic disease who used imatinib subjectively described the most frequent symptoms as being periorbital oedema, nausea, fatigue, exhaustion, cognitive impairment, muscle pain and cramps, and joint pain. Patients also described the considerable impact of these symptoms on their daily lives, again pointing out the gap between physician-reported side-effects and the lived experiences of patients. Quantitative data are scarce: one study reported severe fatigue in one-third of GIST patients on TKI [18], while another study reported diarrhoea, fatigue, and insomnia [19].

To date, interventional studies in GIST patients often use generic (e.g. Short-Form Health Survey (SF-36) [20]) or cancer-generic (e.g. EORTC QLQ-C30 [21]) PROMs that do not assess symptoms specific to TKIs. To incorporate TKI-related symptoms in patient-reported outcome measures for GIST patients in future research, more detailed insight into symptom prevalence, relevance, and priority of issues is needed. In the current study, we use two different data sources for patient-reported symptoms, i.e. survey data and data extracted from an online GIST patient forum to examine: (1) the prevalence of symptoms reported by patients; (2) to what extent the issues reported on a patient forum are covered by existing PROMs (i.e. EORTC QLQ-C30 and items from the EORTC Symptom-Based Questionnaire [16]); and (3) the issues that should be prioritized for incorporation in future HRQoL assessment based on the top 10 most prevalent issues.

## Methods

### Study design and participants

A cross-sectional population-based survey study was conducted among patients aged ≥ 18 years at diagnosis registered in the Netherlands Cancer Registry (NCR) and who had been diagnosed with GIST (according to the ICD-10-GM codes C15-20, C26, C48, and C80), between January 1, 2008, and December 31, 2018. Only patients diagnosed within one of the GIST expertise centres (Radboud University Medical Centre [Nijmegen], Erasmus MC Cancer Institute [Rotterdam], Leiden University Medical Centre, The Netherlands Cancer Institute [Amsterdam], and University Medical Centre Groningen) were selected. Patients who had cognitive impairment or were too ill at the time of the study, according to the advice from their (former) treating specialist, or died prior to the start of the study (according to data from the hospital of diagnosis and/or data from the Dutch municipal personal records database) were excluded. The NCR is a population-based registry which is maintained by the Netherlands Comprehensive Cancer Organisation (IKNL) and collects records, including patient, tumour, and treatment characteristics, on all newly diagnosed cancer patients in the Netherlands based on data from the Nationwide Network and Registry of Histo- and Cytopathology (PALGA) in the Netherlands [22].

Data from the (at the time) public Facebook group of GIST Support International (GSI) was used to automatically extract symptoms from the messages on the patient forum. GSI is a US-based non-profit corporation founded in 2002 [23]. The main aims of the organization are to connect GIST patients and their families and friends, to provide information, and to stimulate research. Members are encouraged to interact and share ideas and experiences in the online community. The forum was moderated by assigned, experienced GSI members.

Ethical approval for the cross-sectional study was provided by the medical ethical committee of the Radboud University Medical Centre (2019–5888). According to the Dutch law, approval of one ethical committee for questionnaire research is valid for all participating centres. Permission to use data from the Facebook group was given by GSI. Discussions were pseudonymized and messages could not be traced back to individual members. No formal approval was needed for the use of data from the public Facebook group, as the General Data Protection Regulation (GDPR) allows the use of data from publicly accessible forums with justified cause.

### Recruitment and data collection

#### Survey study

Eligible patients received an invitation letter from their (ex-)treating physician explaining the goals and procedure of the study. Participants provided informed consent, including permission to link survey data with data from the NCR. Data was collected from September 2020 through June 2021. Survey administration was done within the Patient-Reported Outcomes Following Initial treatment and Long-term Evaluation of Survivorship (PROFILES) registry [24]. PROFILES is a data management system set up in 2009 in the Netherlands for the study of the physical and psychosocial impact of cancer and its treatment. PROFILES contains a large web-based component and is linked directly to clinical data from the NCR. Participants could complete the survey online or on paper upon request.

#### Forum study

The English messages from the patient forum were collected on November 1, 2020, and ranged from October 24, 2009, to November 1, 2020. The number of messages was 125,161 in 14,631 conversational threads. A software pipeline was developed to first extract words containing side-effects from each forum message and then to automatically determine which side-effect is being mentioned. These algorithms were trained on data hand-labelled by human annotators. The sensitivity or recall of the extraction of side-effects is 0.739 meaning 73.9% of the side-effects reported on the forum can be found by the algorithm. The precision is 0.695, which means that 69.5% of the side-effects identified by the algorithm are side-effects. The remaining 30.5% are false positives. The accuracy of automatic labelling of side-effects with SNOMED-CT concepts is 0.645 (i.e. 64.5% of the side-effects are automatically linked to the correct concept in SNOMED-CT) [25]. Text about imatinib was extracted from the forum data as well and then linked to the symptom mentioned in the message that it was most likely associated with. The methods of sensitivity and accuracy analysis, text-extraction, and linkage of the symptom to imatinib are described in detail elsewhere [25].

### Study measures

Questionnaires and individual items from the EORTC Quality of Life Group (QLG) portfolio were selected as they belong to the most frequently used cancer-specific PROMs worldwide and were developed following well-established guidelines [26]. From the 30-item questionnaire EORTC QLQ-C30, version 3.0 [21], 11 symptom-specific items were evaluated (i.e. dyspnoea, pain, feeling weak, appetite loss, nausea, vomiting, constipation, diarrhoea, fatigue, problems with concentrating, and problems with remembering things). Other symptoms related to TKI use were assessed by 8 additional items from the EORTC Symptom-Based Questionnaire (EORTC-SBQ), a 61-item set that was recently developed for patients receiving targeted therapy [16] (i.e. swelling of the face or around the eyes, swelling in any part of the body, muscle aches, pains, or cramps, aches or pains in joints, food and drink tasting different from usual, pain or soreness in mouth, indigestion or heartburn, skin problems). Furthermore, one item about hand-foot syndrome was added from the EORTC Item Library. The items were selected based on prevalence reported in a systematic review of the symptoms associated with TKIs used in the treatment of GIST [27]. One item of own design about the impact of changed physical appearance was added as this was an issue that physicians frequently heard from patients, based on symptoms such as periorbital oedema and hair discolouration.

### Statistical analysis

#### Survey study

For analysis, only patients using TKI at the time of study participation were selected. In the case of low numbers of patients using a specific TKI, the results were only exploratively compared and presented separately in Appendix 1. Prevalence scores for symptoms were determined based on a score of 2 or higher on the 4-point Likert scale being 1 ‘not at all’, 2 ‘a little’, 3 ‘quite a bit’, and 4 ‘very much’, and represented by numbers and percentages out of the total number of patients taking the specific TKI. All analyses were conducted using SPSS version 25.0 (Statistical Package for Social Sciences, Chicago, IL, USA).

#### Forum study

To reduce noise, only side-effects that are mentioned at least five times are included, duplicate side-effects from the same forum message were excluded, and false positives are reduced by excluding cases where no drug is mentioned in the conversational thread [25]. Prevalence of symptoms in the patient forum data was based on how often the symptom was mentioned. Percentages out of the total number of symptoms for each TKI were calculated and reported elsewhere [28].

As a secondary analysis, the 10 most prevalent symptoms for each TKI in the survey study and the forum study were compared based on relative reporting rate. Comparison based on absolute prevalence in the two studies was not possible, because of the difference in measurement.

## Results

### Participants

In the cross-sectional survey study, a total of 521 (former) GIST patients were invited to participate and 328 (response rate 63%) consented and completed the survey. One hundred seven GIST patients used TKI at the time of study participation: 92 used imatinib, 6 sunitinib, 6 regorafenib, and 3 ripretinib. Based on these numbers, we focused on imatinib treatment for this analysis, and results of the explorative analysis for the other TKIs are included in Appendix 1. Characteristics of patients using imatinib are shown in Table 1. No patient characteristics are available from the forum study.

**Table 1:** Patient characteristics from the survey study

	Imatinib (*n* = 92)
Age (mean ± SD)	66.5 ± 10.0
Time since diagnosis in years (mean ± SD)	6.0 ± 2.9
Sex	
Male Female	50 42
Highest formal education Primary school only High school College or university	1 missing 4 (4.4%) 20 (22%) 67 (72.9%)
Relationship status	
Single Married/relationship Separated/divorced Widowed	6 73 6 7
Comorbidities^#^	
None One Two or more	28 17 47
Comorbidities (specified)^#^	
Heart disease Stroke Hypertension Lung disease Diabetes Ulcer or stomach disease Kidney disease Liver disease Anemia or other blood diseases Thyroid disease Depression Osteoarthrosis Back pain Rheumatoid arthritis or other joint inflammation Other cancer	9 2 21 7 7 3 5 7 13 4 8 26 26 6 4

^#^Assessed using the Self-Administered Comorbidity Questionnaire[29]

### Prevalence scores

Prevalence scores for symptoms related to imatinib are shown in Table 2. In the survey study, the three most prevalent patient-reported symptoms for imatinib were fatigue (73%), muscle pain or cramps (73%), and swelling in the face or around the eyes (59%). In the forum study, for imatinib, the three most prevalent were symptoms fatigue (8.6%), nausea (7.8%), and cramp (6.9%).

**Table 2:** Prevalence* scores for symptoms for imatinib

Symptoms	Prevalence* (%)
Survey study (*n* = 92)	
Fatigue	66 (73)
Muscle aches, pains, or cramps	66 (73)
Swelling of the face or around the eyes	54 (59)
Aches or pains in joints	48 (52)
Problems with remembering things	47 (52)
Skin problems (e.g. itchy skin, dry skin, skin discolouration)	46 (50)
Diarrhoea	46 (50)
Feeling weak	38 (41)
Indigestion or heartburn	37 (40)
Swelling in any part of the body	35 (38)
Shortness of breath	31 (37)
Food and drink tasting different from usual	33 (36)
Pain	31 (34)
Problems with concentrating	29 (32)
Problems because of changed appearance	28 (30)
Appetite loss	21 (23)
Nausea	21 (23)
Hand-foot syndrome	20 (22)
Pain or soreness in mouth	16 (17)
Constipation	11 (12)
Vomiting	5 (5)
Forum study (10 most prevalent symptoms adapted from https://dashboard-gist-adr.herokuapp.com/ accessed on July 14, 2021)	
Fatigue	1181 (8.6)
Nausea	1062 (7.8)
Cramp	939 (6.9)
Disorder of skin	680 (5.0)
Oedema	544 (4.0)
Pain^a^	524 (3.8)
Alopecia	466 (3.4)
Altered bowel function^b^	433 (3.2)
Pain in limb^c^	325 (2.4)
Facial swelling	235 (1.7)

^*^For the survey data, prevalence is based on the percentage of patients with this symptom out of the total number of patients taking imatinib. For the forum data, prevalence is based on percentages of each symptom out of the total number of symptoms for imatinib

^a^Includes chronic pain and generalized aches and pains

^b^Includes constipation and diarrhoea

^c^Includes any pain in the upper or lower limb, excludes cramp, muscle pain, hand-foot syndrome

### Relation between questionnaire and forum symptoms

Table 3 shows the coverage of the 10 most reported symptoms related to imatinib on the online forum in the EORTC QLQ-C30, the EORTC-SBQ, and the EORTC Item Library. The EORTC QLQ-C30 includes 4 out of 10 most prevalent symptoms on the online forum. The EORTC-SBQ and EORTC Item Library cover 9 and 10 symptoms, respectively.

**Table 3:** Coverage of symptoms from online forum in questionnaires

Symptoms from forum	EORTC QLQ-C30	EORTC-SBQ	EORTC Item Library
Fatigue	X	X	X
Nausea	X	X	X
Cramp		X	X
Disorder of skin		X	X
Oedema		X	X
Pain	X		X
Alopecia			X
Altered bowel function	X (diarrhoea, constipation)	X	X
Pain in limb		X	X
Facial swelling		X	X

Finally, the 10 most prevalent symptoms in the survey study and the forum study were compared based on relative reporting rate, indicated as in descending values in Table 4. For imatinib, 7 symptoms overlapped between the two studies. Symptoms from the forum study that were not in the top 10 for imatinib in the survey study were nausea, pain, and alopecia (Table 4). Fatigue was the most prevalent symptom both in the survey study and the forum study, but the relative reporting rates for the other symptoms differed. Due to the very low number of patients taking sunitinib, regorafenib, or ripretinib in the survey studies, no formal comparison was made. But explorative analysis showed a similar pattern of overlap between the 10 most prevalent symptoms of the two studies (Appendix 1).

**Table 4:**
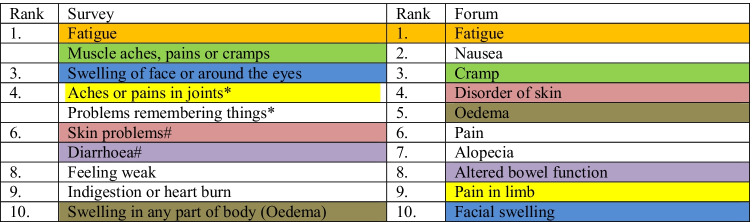
Ranking in descending values of prevalence of symptoms related to imatinib in survey study and forum study. *Same prevalence (52%); #same prevalence (50%)

## Discussion

This paper describes the use of two sources for patient-reported symptom rates outside trials in GIST patients treated with imatinib: surveys and messages from an online patient forum. The most prevalent symptoms in both studies were fatigue and muscle pain or cramps. The EORTC-SBQ and EORTC Item Library cover the majority of symptoms out of the top 10 most prevalent symptoms on the online forum, but coverage by the EORTC QLQ-C30 was limited. More than half of the 10 most prevalent symptoms were shared between the two sources, but the relative reporting rate of symptoms differed. The prevalent symptom from the online forum that was not covered by the EORTC-SBQ was alopecia. A similar pattern was found for other TKIs prescribed for GIST in the explorative analysis.

The symptoms found in the survey and the forum study mirror the side-effect profiles of imatinib reported in the registration trials, but relative reporting rates differ for example for muscle cramps [8]. These symptoms occur more frequently over time and may therefore be registered less, or not recognized as adverse drug effects during the initial registration trials. Furthermore, previous work has shown that patients report symptoms earlier and more frequently with worse symptom severity than clinicians [30], and this was particularly the case for muscle cramps and musculoskeletal pain in chronic myeloid leukemia (CML) patients using imatinib [31]. Studies investigating the prevalence of patient-reported symptoms in patients with GIST using TKIs are scarce. Previous studies showed that, similar to our results, severe fatigue is common in GIST patients, especially in those taking TKI [18, 32]. Consequently, fatigue had a negative impact on overall quality of life, functional, psychological, and physical well-being [18]. A study investigating symptom burden with the MD Anderson Symptom Inventory for GISTs (MDASI-GIST) identified the most severe symptoms in GIST patients, including muscle soreness and cramping, fatigue, and general weakness [33], matching the most prevalent symptoms found in our data. Unfortunately, the MDASI-GIST is not validated outside the USA. Symptoms that were most prevalent in our study are also the same as the self-reported side-effects in a qualitative study, such as muscle pain, cramps, and oedema for imatinib [17].

This paper demonstrates that the EORTC portfolio adequately captures what is important to patients on TKI treatment regarding symptoms and HRQoL, although the cancer-generic EORTC QLQ-C30 on its own lacks most treatment-specific symptoms that were reported on the forum. The forum data also reveals side-effects that are not routinely included in PRO-assessment for TKIs, i.e. alopecia. Although it is usually less extensive than in chemotherapy, alopecia is a known adverse effect of TKIs [34, 35] and is more prolonged given the continuous daily dosing schedule. The fact that the reporting rate of alopecia is high on the patient forum indicates that it is an important symptom for patients taking TKIs nonetheless, and can be considered for inclusion HRQoL assessment in future studies.

Differences in relative reporting rate between the two data sources are difficult to interpret, because details on patient characteristics and clinical information were lacking. For example, nausea was ranked higher in the forum study for imatinib treatment than in the survey study. Nausea most frequently occurs in the beginning of TKI treatment, and declines over time, e.g. with the use of anti-emetics or changes in dosing schedules [8]. As the survey study included patients who were at least 2.5 years since diagnosis at the time of participation, we hypothesize that the presence of nausea may have already declined whereas patients posting on the forum about nausea may just have started treatment. Furthermore, one might hypothesize that patients who post messages or complete questionnaires experience more symptoms or higher impact on HRQoL than those that do not; however, data on the symptom burden or HRQoL of patients causing them to be active in online cancer communities is scarce. Ector et al. [36] reported that TKI treatment itself and QoL were not associated with a need for more or less information in chronic myeloid leukemia patients. One study found no differences in the use of online support groups for arthritis, fibromyalgia, and breast cancer between patients who post messages and patients who only read messages in case they experienced many or new symptoms [37]. Comparison with a population that was not active on online support groups is not available. The currently used survey study in Dutch GIST patients included evaluation of social media use to investigate differences between patients that use social media to conversate with other patients and those that do not. Analysis of these data is currently ongoing.

Some limitations need to be taken into consideration. First, online forum data and questionnaire data are unavoidably subject to sample bias [38, 39] and responder bias, respectively. However, as no background information is available for the posters on the online forum, we cannot assess bias in the current analysis. Furthermore, we have no data on which and how many symptoms were reported by family members of GIST patients who also had access to the forum. In recent years, the use of online support groups by family members was not significantly different from cancer survivors [40], which could also apply to our forum data. Assessment of responder bias in the survey study was also not possible for the subgroup of patients using TKI included in the current analysis, because information about TKI treatment was not available for the non-responder population. Second, a formal comparison of symptom prevalence and prioritization between the two datasets was not possible because of the difference in measurement. The survey study only assessed a limited number of predefined symptoms, whereas the forum study used uncensored, unsolicited reports resulting in a larger number of different symptoms [25]. Prevalence rates were also calculated differently from the two sources, in which methods for extraction of symptoms and linkage to TKI from the online forum could also have induced false positives, e.g. by extracting text that in fact did not refer to a symptom or linkage of a symptom to the wrong TKI [25]. Additionally, patients might post about the same symptom more than once, which could not be assessed without assessing user names and breaching privacy, causing a skewed distribution in the actual frequency and relative reporting rate of the symptoms. Third, it remains challenging to distinguish for patients, and therefore for researchers as well, if symptoms are solely related to treatment, or to tumour burden or comorbidities [41]. This could be clarified in future studies by asking patients to consider the time of onset, or improvement after dose modification. Fourth, the number of patients taking other TKIs than imatinib was low in the survey study, limiting generalizability. This is probably due to including patients who were at least 2.5 years since diagnosis, selecting patients with a favourable course of disease, and/or response to imatinib. Lastly, insufficient information was available in this study to prioritize symptoms for specific subgroups based on clinical characteristics such as time since the start of TKI treatment and treatment setting (adjuvant or palliative).

This study presents an innovative approach to gain more insight into patient-reported symptoms in GIST patients using TKI. Using automatic extraction of symptoms from an online patient forum and linking them to specific TKIs offers a valuable complementary resource for PRO-data. In addition to interviews with patients and health care professionals that are the primary sources for HRQoL issues in PROMs, forum data may include the perspective of patients who would not be invited or not willing to participate in such interviews. It provides insight into which symptoms are relevant in a large group of patients, which is uncommon for rare cancers, which may help prioritize the selection of HRQoL issues for evaluation (e.g. the high prevalence of muscle cramps in this study). Lastly, forum data raises symptoms or side-effects that are not part of existing PROMs (i.e. alopecia in this study), prompting further investigation whether or not they can be included in PROMs and keeping PROMs up to date. This approach is compatible with the novel flexible strategy for HRQoL assessment by the EORTC QLG, combining existing EORTC questionnaires with add-on symptom questions from the EORTC Item Library [42, 43]. In studies investigating GIST (and possibly other cancer) patients using TKIs, we recommend combining the EORTC QLQ-C30 (to facilitate comparison of cancer-generic HRQoL issues between studies and other (cancer-)populations) with a selection of symptoms from the EORTC-SBQ and individual items from the EORTC Item Library (for symptoms that are missing in the EORTC-SBQ). In studies where only symptoms or adverse events are of interest, the Patient-Reported Outcomes version of the Common Terminology Criteria for Adverse Events (PRO-CTCAE) can also be used [44]. In clinical practice, symptoms can be selected based on known side-effects from registration trials and clinical experience. Hierarchy in relevance may be based on data from patient forums. More sensitive detection and measurement of symptoms and their impact on HRQoL will help improve assessment of treatment outcomes in research and shared decision-making about (dis-)continuation of treatment in clinical practice. In conclusion, this study shows the prevalence of TKI treatment–related symptoms reported by GIST patients in a survey and on an online patient forum in a real-life setting. Frequently reported symptoms were not fully covered by cancer-generic measures, and additional issues were reported on the patient forum. Combining these sources of patient-reported data creates a more comprehensive overview of symptom experience and treatment side-effects in GIST patients and helps improve future HRQoL assessment in care and research.

## Supplementary Information

Below is the link to the electronic supplementary material.

Supplementary file1 (DOCX 26 KB)

## Data Availability

The datasets generated and/or analysed during the survey study are not publicly available due to them containing information that could compromise research participant consent but are available from the corresponding author on reasonable request. The dataset from the forum study is publicly available via https://dashboard-gist-adr.herokuapp.com/.

## Code availability

For the forum study, all technical details of the pipeline are reported in a separate publication [25]. The code will be made available upon publication.
