# Supplementary material for: Symptoms reported by gastrointestinal stromal tumour (GIST) patients on imatinib treatment: combining questionnaire and forum data
Source: Support Care Cancer. 2022 Mar 2;30(6):5137–46. doi: 10.1007/s00520-022-06929-3 (PMC9046353; doi:10.1007/s00520-022-06929-3)
Supplement: Supplementary file 1 — Supplementary file1 (DOCX 26 KB) [file 520_2022_6929_MOESM1_ESM.docx]

**Supplementary material**

*Patient characteristics from the survey study*

|  | Sunitinib (n=6) | Regorafenib (n=6) | Ripretinib (n=3) |
| --- | --- | --- | --- |
| Age (mean± SD (range)) | 74.4 ± 8.0 (64-86) | 65.5 ± 4.3 (60-71) | 64.9 ± 4.6 (60-69) |
| Time since diagnosis in years (mean± SD (range)) | 6.0 ±2.1 (3.8-9.4) | 5.8 ± 1.8 (3.8 – 8.2) | 3.4 ± 1.2 (2.1-4.6) |
| Sex   - Male - Female | 2  4 | 5  1 | 2  1 |
| Highest formal education   - Primary school only - High school - College or university | 0  1 (16.7%)  5 (83.3%) | 1 (16.7%)  2 (33.3%)  3 (50%) | 0  0  3 (100%) |
| Relationship status   - Single - Married/relationship - Separated/divorced - Widowed | 0  4  0  2 | 0  5  0  1 | 0  3  0  0 |
| Comorbidities   - None - One - Two or more | 2  1  3 | 3  2  1 | 1  1  1 |

*Prevalence scores for symptoms for different tyrosine kinase inhibitors (TKI)*

|  | Sunitinib (n=6) | Regorafenib (n=6) | Ripretinib (n=3) |
| --- | --- | --- | --- |
| Symptoms | *Prevalence (%) | *Prevalence (%) | *Prevalence (%) |
| SURVEY STUDY | | | |
| Swelling of the face or around the eyes | 3 (50) | 2 (33) | 0 (0) |
| Swelling in any part of the body | 1 (18) | 1 (18) | 0 (0) |
| Muscle aches, pains, or cramps | 4 (67) | 4 (67) | 3 (100) |
| Aches or pains in joints | 4 (67) | 2 (33) | 1 (33) |
| Food and drink tasting different from usual | 5 (83) | 4 (67) | 0 (0) |
| Pain or soreness in mouth | 5 (83) | 2 (33) | 0 (0) |
| Indigestion or heartburn | 5 (83) | 1 (18) | 1 (33) |
| Skin problems (e.g. itchy skin, dry skin, skin discolouration) | 5 (83) | 4 (67) | 2 (67) |
| Hand-foot syndrome | 3 (50) | 3 (50) | 0 (0) |
| Problems because of changed appearance | 2 (33) | 0 (0) | 3 (100) |
| Feeling confused | 1 (18) | 1 (18) | 0 (0) |
| Trouble speaking | 2 (33) | 1 (18) | 0 (0) |
| Auditory hallucinations | 0 (0) | 0 (0) | 0 (0) |
| Visual hallucinations | 1 (18) | 0 (0) | 0 (0) |
| Shortness of breath | 1 (18) | 4 (67) | 1 (33) |
| Pain | 4 (67) | 3 (50) | 2 (67) |
| Feeling weak | 5 (83) | 4 (67) | 1 (33) |
| Appetite loss | 4 (67) | 2 (33) | 1 (33) |
| Nausea | 5 (83) | 1 (18) | 0 (0) |
| Vomiting | 2 (33) | 1 (18) | 0 (0) |
| Constipation | 3 (50) | 1 (18) | 2 (67) |
| Diarrhoea | 4 (67) | 2 (33) | 1 (33) |
| Fatigue | 6 (100) | 5 (83) | 3 (100) |
| Problems with concentrating | 3 (50) | 1 (18) | 1 (33) |
| Problems with remembering things | 3 (50) | 2 (33) | 1 (33) |
| FORUM STUDY (*adapted from:* <https://dashboard-gist-adr.herokuapp.com/> *accessed on July 14, 2021)* | | | |
| Fatigue | 184 (8.0%) | 117 (9.5%) | 40 (12.6%) |
| Nausea | 111 (4.8%) | 35 (2.8%) | 14 (4.4%) |
| Cramp | 32 (1.4%) | 30 (2.4%) | 14 (4.4%) |
| Disorder of skin | 59 (2.6%) | 36 (2.9%) | 12 (3.8%) |
| Oedema | - | - | - |
| Pain^a^ | 92 (4.0%) | 80 (6.5%) | 13 (4.1%) |
| Alopecia | 90 (3.9%) | 72 (5.8%) | 42 (13.4%) |
| Altered bowel function^b^ | 121 (5.2%) | 42 (3.4%) | 5 (1.6%) |
| Pain in limb^c^ | 137 (5.9%) | 87 (7.1%) | 13 (4.1%) |
| Facial swelling | - | - | - |
| Painful mouth | 142 (6.1%) | 27 (2.2%) | - |
| Weight loss | 20 (0.9%) | 38 (3.1%) | 6 (1.9%) |
| Hand-foot syndrome | 27 (1.2%) | 58 (4.7%) | 10 (3.1%) |
| Hypertensive disorder | 86 (3.8%) | - | 26 (2.1%) |
| Taste sense altered | 77 (3.3%) | - | - |

^*^For the survey data, prevalence is based on percentage of patients with this symptom out of the total number of patients taking each TKI. For the forum data, prevalence is based on percentages of each symptom out of the total number of symptoms for each TKI were calculated.

^a^includes: chronic pain and generalized aches and pains

^b^includes: constipation and diarrhoea

^c^includes: any pain in upper or lower limb, excludes: cramp, muscle pain, hand-foot syndrome

Ripretinib was not included the exploration of relation between questionnaire and forum symptoms due to the very low number of patients taking this TKI in the survey study.

*Ranking of prevalence of symptoms related to sunitinib in survey study and forum study*

| Rank | Survey | Rank | Forum |
| --- | --- | --- | --- |
| 1. | Fatigue | 1. | Fatigue |
| 2. | Pain or soreness in mouth* | 2. | Painful mouth |
|  | Indigestion or heart burn* | 3. | Pain in limb |
|  | Skin problems * | 4. | Altered bowel function |
|  | Nausea* | 5. | Nausea |
|  | Food and drink tasting different from usual* | 6. | Pain |
|  | Feeling weak* | 7. | Alopecia |
| 8. | Muscle aches, pains or cramps # | 8. | Hypertensive disorder |
|  | Aches and pains in joints# | 9. | Taste sense altered |
|  | Pain# | 10. | Disorder of skin |
|  | Appetite loss # |  |  |
|  | Diarrhea# |  |  |

* same prevalence (83%) # same prevalence (67%)

*Ranking of prevalence of symptoms related to regorafenib in survey study and forum study*

| Rank | Survey | Rank | Forum |
| --- | --- | --- | --- |
| 1. | Fatigue | 1. | Fatigue |
| 2. | Muscle aches, pains or cramps* | 2. | Pain in limb |
|  | Shortness of breath* | 3. | Pain |
|  | Skin problems * | 4. | Alopecia |
|  | Feeling weak* | 5. | Hand-foot syndrome |
|  | Food and drink tasting different from usual* | 6. | Altered bowel function |
| 7. | Hand-foot syndrome# | 7. | Weight loss |
|  | Pain # | 8. | Disorder of skin |
| 9. | ‡ | 9. | Nausea |
|  |  | 10. | Cramp |

* same prevalence (67%) # same prevalence (50%) ‡ six symptoms with same prevalence (33%)
